# Human Granulocytic Anaplasmosis, South Korea, 2013

**DOI:** 10.3201/eid2010.131680

**Published:** 2014-10

**Authors:** Kye-Hyung Kim, Jongyoun Yi, Won Sup Oh, Nak-Hyun Kim, Su Jin Choi, Pyoeng Gyun Choe, Nam-Joong Kim, Jong-Koo Lee, Myoung-don Oh

**Affiliations:** Seoul National University College of Medicine, Seoul, South Korea (K.-H. Kim, N.-H. Kim, S.J. Choi, P.G. Choe, N.-J. Kim, J.-K. Lee, M.-D. Oh);; Pusan National University School of Medicine, Busan, South Korea (J. Yi);; Kangwon National University School of Medicine, Chuncheon, South Korea (W.S. Oh)

**Keywords:** human granulocytic anaplasmosis, HGA, Anaplasma phagocytophilum, bacteria, ticks, vector-borne infections, zoonoses, severe fever with thrombocytopenia syndrome, SFTS, South Korea

## Abstract

We report a patient with human granulocytic anaplasmosis in South Korea. The patient had fever and thrombocytopenia. Human granulocytic anaplasmosis was confirmed by seroconversion, PCR, and sequence analysis for *Anaplasma phagocytophilum*. Morulae were observed in the cultured HL-60 cells inoculated with blood from the patient.

*Anaplasma phagocytophilum*, the causative agent of human granulocytic anaplasmosis (HGA), is a zoonotic tickborne pathogen transmitted by ixodid ticks that infects wild and domestic mammals and humans (*1**–**3*). HGA was first identified in the United States in 1994 (*1*) and subsequently in countries in Europe (*3*), China (*4*), and Japan (*5*).

To our knowledge, there is no report regarding the clinical description of HGA patients in South Korea. However, *A. phagocytophilum* has been detected in *Haemaphysalis longicornis*, *Ixodes nipponensis*, and *I. persulcatus* ticks (*6**,**7*) in this country. Molecular epidemiologic studies detected *A. phagocytophilum* in 2.6% (5/196) of striped field mice (*7**,**8*) and in 63.6% (42/66) of Korean water deer (*9*). Seroprevalence studies showed that 1.8% of serum samples from patients with acute fever were positive for *A. phagocytophilum* by an immunofluorescence antibody test (*10*). We report a patient with HGA and characterized the *A. phagocytophilum* isolate from this patient.

## The Study

On May 17, 2013 (day 0, day of illness onset), fever, chills, nausea, and vomiting developed in a 57-year-old woman who lived in Chuncheon, Gangwon Province, South Korea. She came to a local clinic on the same day, and treatment with antipyretic drugs was initiated. The patient reported being bitten by a tick on her right shoulder while mountain climbing in Gangwon Province 5 days before the fever occurred. She did not recall having contact with any domestic animals and had no history of travel outside South Korea in the month before illness onset.

On day 4, the patient came to Kangwon National University Hospital (Chuncheon, South Korea) with a persistent fever despite use of antipyretics. The patient had a temperature of 39.2°C, blood pressure of 76/61 mm Hg, heart rate of 88 beats/min, and an oxygen saturation level of 98.3% on room air. An erythematous maculopapular lesion, 3 cm in diameter, was observed around the tick bite on the right shoulder. Laboratory tests showed pancytopenia and increased serum aminotransferase levels (Table).

**Table:** Laboratory findings for patient with human granulocytic anaplasmosis, South Korea, 2013*

Laboratory test (reference range)	Day 5†	Day 6	Day 7	Day 8	Day 9	Day 10	Day 11	Day 19‡
Leukocytes, cells/μL (3,800–10,000)	2,400	1,100	7,400	5,800	5,200	5,700	4,800	5,700
Neutrophils, % (40–70)	91	28	79	62	50	42	39	51
Lymphocytes, % (20–50)	7	64	17	31	39	45	47	42
Hb, g/dL (12.3–15.3)	9.4	9.3	9.4	8.7	8.8	10.4	9.8	10.3
Hct, % (36.6–44.2)	28.5	27.3	28.8	26.4	27.4	32.5	30.5	31.0
Platelets/μL (140,000–400,000)	78,000	61,000	63,000	69,000	105,000	194,000	249,000	348,000
AST, IU/L (15–41)	137	179	80	48	106	111	64	25
ALT, IU/L (14–54)	60	93	73	55	84	105	83	26
CPK, IU/L (38–234)	74	156	148	64	41	31	NA	39
LDH, IU/L (100–190)	280	384	275	203	243	239	180	134
Creatinine, mg/dL (0.4–1.0)	0.8	0.7	0.5	0.5	0.5	0.7	NA	0.6
C-reactive protein, mg/dL (0–0.50)	6.11	7.04	NA	NA	NA	NA	0.62	NA
PT, INR (0.92–1.17)	1.05	1.17	1.00	0.97	NA	NA	NA	NA
aPTT, s (31.0–43.7)	44.8	57.6	49.3	41.5	NA	NA	NA	NA
Fibrinogen, mg/dL (207–408)	NA	304	328	308	NA	NA	NA	NA

*Day 0 was day of illness onset. Hb, hemoglobin; Hct, hematocrit; AST, aspartate aminotransferase; ALT, alanine aminotransferase; CPK, creatine phosphokinase; NA, not available; LDH, lactate dehydrogenase; PT, prothrombin time; INR, international normalized ratio; aPTT, activated partial thromboplastin time. †Blood samples for culture and for immunofluorescence antibody test were obtained. ‡Blood samples for immunofluorescence antibody test were obtained.

On day 5, the patient was treated with norepinephrine, ceftriaxone, and doxycycline. On day 7, her blood pressure and temperature were within reference ranges and a norepinephrine infusion was discontinued. Peripheral blood smear showed no evidence of malaria, and antibody titer against *Orientia tsutsugamushi* was <1:40. Test results for antibodies against Hantaan virus, leptospira, *Borrelia burgdorferi*, and *Coxiella burnetii* were negative. Blood cultures prepared at the time of the visit were negative. On day 11, she was discharged from the hospital, and treatment with antimicrobial drugs was discontinued.

Because symptoms and laboratory findings, such as thrombocytopenia, anemia, and increased aminotransferase levels (Table), of the patient were similar to those for severe fever with thrombocytopenia syndrome (SFTS), anticoagulated blood and serum were obtained on day 5 before treatment with doxycycline and on day 19. Samples were sent to Seoul National University College of Medicine for additional diagnostic tests.

RNA was extracted from blood by using the QIAamp Viral RNA Mini Kit (QIAGEN, Hilden, Germany). Reverse transcription PCR for SFTS virus was performed according to described methods (*11*) and showed negative results. No cytopathic effect was observed in Vero cells and DH82 cells after inoculating them with a blood sample.

DNA was extracted from the blood by using the QIAamp DNA Mini Kit (QIAGEN) to detect *A. phagocytophilum* 16S rRNA gene, *ankA*, *groESL* operon, and *msp2*. Nested PCR was conducted to amplify a 926-bp fragment of the 16S rRNA gene by using an *A. phagocytophilum* species-specific primer set (*12*). Direct sequencing of the PCR product was performed to confirm *A. phagocytophilum*, and the partial 16S rRNA gene sequence was deposited in GenBank (accession no. KF805344).

A nucleotide basic local alignment search tool (BLAST) search (http://blast.ncbi.nlm.nih.gov/Blast.cgi) with the obtained sequence showed matches to only *A. phagocytophilum* sequences (99.3%–100% similarities). Aligning ≥2 sequences by using BLAST showed that the obtained sequence had lower similarities (94.3%–98.6%) with other *Anaplasma* species (*A. marginale* [GenBank accession no. NR_074556], *A. centrale* [NR_074356], *A. ovis* [AY262124], *A. platys* [EF139459], and *A. bovis* [HM131218]) and with *Ehrlichia* species (*E. chaffeensis* [NR_037059], *E. ewingii* [NR_044747], and *E. canis* [NR_074386]).

**Figure 1:**
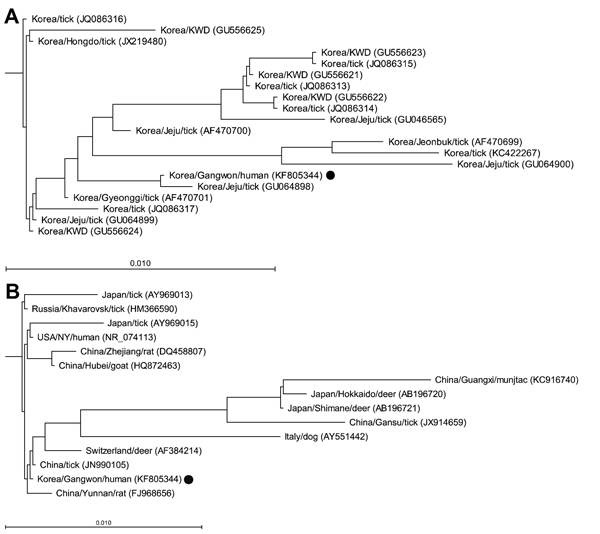
Phylogenetic trees for partial 16S rRNA gene sequences of an *Anaplasma phagocytophilum* isolate obtained from a patient with human granulocytic anaplasmosis in South Korea (black dots) and those of the *A. phagocytophilum* strains reported from A) South Korea and B) other countries. Trees were constructed by using the neighbor-joining method. Locations (country/province or city), hosts, and GenBank accession numbers are indicated. Branch lengths of trees show evolutionary distances. Scale bars indicate 1.0% sequence distance. KWD, Korean water deer.

Comparison of the 16S rRNA gene sequence with sequences for other *A. phagocytophilum* strains from South Korea showed that this sequence was relatively distant from those of Korean water deer isolates, but similar to those of tick isolates from Jeju Island (Figure 1, panel A). Comparison of the 16S rRNA gene sequence with other sequences of *A. phagocytophilum* strains reported from other countries showed that our isolate was relatively distant from the strains obtained from ticks collected in Gansu and Guangxi, China, and Hokkaido and Shimane, Japan. The isolate was similar to a human isolate from the United States, tick isolates from Russia and China, and animal isolates from Yunnan, Zhejiang, and Hubei, China (Figure 1, panel B).

Nested PCR was performed to amplify a 667-bp fragment of the *ankA* gene (*13*), and single non-nested PCRs were performed to amplify a 1,715-bp fragment of the *groESL* operon (*14*) and a 334-bp fragment of the *msp2* gene (*15*). Direct sequencing of PCR products was performed to confirm *A. phagocytophilum*, and partial *ankA*, *groESL*, and *msp2* sequences were deposited in GenBank (accession nos.KJ677106–KJ677108). Nucleotide BLAST searches with obtained sequences showed matches to only *A. phagocytophilum* sequences: 94.1%–97.9% similarities for *ankA*, 97.9%–99.5% for *groESL*, and 97.8%–99.6% for *msp2*.

**Figure 2:**
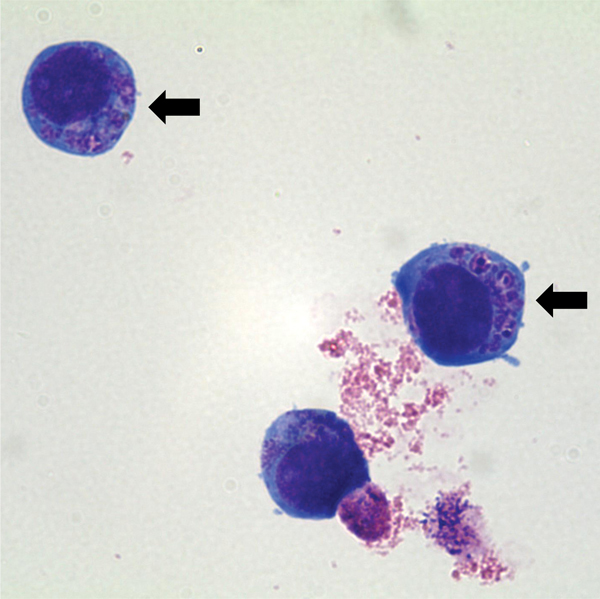
Light micrograph of *Anaplasma phagocytophilum* cultured in human promyelocytic cell line HL-60, showing *A. phagocytophilum* morulae as basophilic and intracytoplasmic inclusions (arrows). Wright–Giemsa stain, original magnification x1,000.

For *A. phagocytophilum* culturing, a suspension of human promyelocytic cell line HL-60 (ATCC CCL-240) was inoculated with the patient’s blood sample (day 5 postillness, before treatment with antimicrobial drugs) and incubated at 37°C in an atmosphere of 5% CO_2_ (*10*). The culture suspension was examined microscopically by Wright-Giemsa staining of cytocentrifuged preparations of cells at 2 to 3-day intervals. Subculture was performed on day 14 postinoculation. On day 8 post-subculture, morulae within cultured HL-60 cells were observed (Figure 2).

Nested PCRs to amplify 16S rRNA and *ankA* genes showed positive results for isolates from day 14 postinoculation and from day 10 postsubculture. Single, non-nested PCRs to amplify *groESL* and *msp2* genes showed positive results for the isolate from day 14 postinoculation but negative for the isolate from day 10 postsubculture. Comparison of sequences from culture isolates and those from PCR products directly amplified from the blood sample showed that these sequences were identical.

For serologic diagnosis, an immunofluorescence antibody test kit for *A. phagocytophilum* (Fuller Laboratories, Fullerton, CA, USA) was used. Positive cutoff titers were 1:16 for IgM and 1:80 for IgG, according to the manufacturer’s instructions. Specific IgM titers increased from 1:16 (day 5) to ≥1:256 (day 19). Specific IgG antibody titers increased from 1:20 (day 5) to 1:160 (day 19).

## Conclusions

We confirmed a case of HGA in a patient in South Korea (who was suspected of having SFTS) by using serologic analysis, PCR, culture, and sequence analysis for *A. phagocytophilum*. According to the case definition of HGA of the US Centers for Disease Control and Prevention (Atlanta, GA, USA) (*2*), this case fulfilled the criteria for anaplasmosis. The patient had a history of a tick bite, and the clinical symptoms and laboratory findings were similar to those of SFTS, an emerging vector-borne disease in South Korea (*11*).

No effective treatment for SFTS is available, but doxycycline is effective for treating HGA (*2*). Clinical features of HGA may be confused with those for SFTS, which may result in inappropriate treatment and severe outcomes. Therefore, not only SFTS but also HGA should be considered as differential diagnoses for patients with fevers and thrombocytopenia after tick bites.
